# Stability of Facial Affective Expressions in Schizophrenia

**DOI:** 10.1155/2012/867424

**Published:** 2012-03-22

**Authors:** H. Fatouros-Bergman, J. Spang, J. Merten, G. Preisler, A. Werbart

**Affiliations:** ^1^Karolinska Institutet, Department of Clinical Neuroscience, Center for Psychiatric Research, 171 77 Stockholm, Sweden; ^2^Department of Psychology, Stockholm University, 106 91 Stockholm, Sweden; ^3^Psychiatric Research Centre, 701 16 Örebro, Sweden; ^4^Universität des Saarland, 66 123 Saarbrücken, Germany

## Abstract

Thirty-two videorecorded interviews were conducted by two interviewers with eight patients diagnosed with schizophrenia. Each patient was interviewed four times: three weekly interviews by the first interviewer and one additional interview by the second interviewer. 64 selected sequences where the patients were speaking about psychotic experiences were scored for facial affective behaviour with Emotion Facial Action Coding System (EMFACS). In accordance with previous research, the results show that patients diagnosed with schizophrenia express negative facial affectivity. Facial affective behaviour seems not to be dependent on temporality, since within-subjects ANOVA revealed no substantial changes in the amount of affects displayed across the weekly interview occasions. Whereas previous findings found contempt to be the most frequent affect in patients, in the present material disgust was as common, but depended on the interviewer. The results suggest that facial affectivity in these patients is primarily dominated by the negative emotions of disgust and, to a lesser extent, contempt and implies that this seems to be a fairly stable feature.

## 1. Introduction

Studies of facial behaviour have shown a clear reduction of facial expressiveness and of facial *affective* expressiveness in patients diagnosed with schizophrenia [1–11]. The reduction of facial expressiveness is especially prominent in the upper face [12] and has also been observed while patients are confronted with emotional stimuli [6, 10, 12, 13] as well as when they are imitating emotional stimuli [14]. Interestingly, most findings point at the conclusion that reduction of facial expressiveness is not correlated with impaired emotional experience [8, 10, 11]. The patients' capacity for emotional recognition is also reduced [11], a very robust finding according to a recent review [15]. However, facial emotional expressiveness is also reduced in patients with depression [6, 12], but patients with schizophrenia are still distinguished in this respect from patients with depression, Parkinson's disease, and right hemisphere brain damage [10] since their diminished expressiveness is more prominent. Furthermore, patients with schizophrenia have been found to limit their facial affective repertoire to mainly negative affective expressions [7, 11, 16], an observation that may be present even before the clinical onset of psychosis [17]. Contempt was found to be the most frequent affect shown by these patients [5, 7, 18] and they showed significantly less happiness compared to healthy subjects [5]. Healthy subjects, on the other hand, interacting with each other, usually show a variety of both negative and positive affects, and the most frequent affect shown by them is usually happiness [16]. Overall facial activity has been reported as 2.3 times higher in interactions amongst healthy subjects compared to healthy subjects interacting with inpatients with schizophrenia [5, 7, 19].

### 1.1. Objectives

The objective was to replicate previous findings regarding negative facial affectivity in schizophrenia and to study the stability of these facial affective expressions. One focus was to examine whether facial affective behavior is dependent on temporality. The other focus was to test whether the patients's affective behavior is person dependent. The following hypotheses were tested.

1. There are no substantial changes in the type of affects that the patients display across several interview occasions.
2. The patient's affects are not altered by the change of interview partner.

## 2. Methods

### 2.1. Subjects

Eight patients diagnosed with schizophrenia according to DSM-IV (1994) agreed to participate in the present study. Patients' characteristics at the onset of the study are presented in Table 1. All the included patients came from a psychiatric clinic with integrated in- and outpatient care, had at least one past inpatient admissions but were not in an acute psychotic state at the time of the interviews and either retired or unemployed. The patients first received a letter with information about the study, and they participated after having given their informed consent. The research project has been approved by the Committee of Ethics, Örebro County Council (605/00, 29/03).

### 2.2. Procedures

#### 2.2.1. The Interviews

Thirty-two interviews were conducted in a clinical setting by two female psychologists both in their early thirties and experienced in working with patients diagnosed with schizophrenia. An open interview model [20] was employed similar to interviews used in clinical settings. The interviews focused on the patients' own problem formulation and background description to their current problems. The interview content centred often on psychotic experiences as well as difficulties in the patient's life. The narratives often included stories about mental suffering and references to the personal crisis stemming from psychotic experiences and hospitalization. The interviewer adjusted the questions to the patient's answers. The interview was designed to encourage mutuality in the interaction and elicit the patient's own thoughts and perspectives. Each patient was interviewed three times by the first interviewer (interview A_1_, interview B_1_, and interview C_1_). The interval between interviews A_1_, B_1_, and C_1_ was one week. When these interviews were finished the patients were interviewed another time by the second interviewer (interview A_2_). Each interview lasted about 45 minutes. Verbatim transcripts of the interviews included pauses and sighing.

#### 2.2.2. Video Recording

The interviews were recorded by three remote-control cameras. One camera focused on the patient's face, one on the interviewer's, and one on the whole scene. The camera uptakes were synchronised and displayed on a single screen provided with time code. These were stored on DVD-discs and replayed including slow motion on a computer using Power-DVD version 3.0 software.

#### 2.2.3. Coding

The Emotion Facial Action Coding System (EMFACS) [21], a coding system for video data, was used to evaluate the patient's and interviewers' facial affective behaviour. EMFACS is based on the Facial Action Coding System (FACS) [22] a coding system for registering all visible facial mimic movements, called Action Units (AUs). Two sequences, 3 minutes each, where the patients were describing their psychotic experiences were selected from each interview. These narratives seemed emotional and were assumed to trigger facial affectivity. First, the interviews were replayed several times and all visible mimic movements (AU) were coded for the patient and interviewer. In a second step the AUs were interpreted with an emotional dictionary software. This software compares the combinations of AUs with the EMFACS dictionary and registers seven affects: anger, contempt (AU 14, unilateral AU 12 or AU 14), disgust (AU 9 or AU 10), sadness, fear, surprise, and happiness (AU 6 + AU 12). Positive affects were operationalised as happiness and surprise and negative affects as contempt, disgust, anger, sadness and fear. When referring to happiness only Duchenne smiles were coded (AU 6+12) and not just social smiles (just AU 12). Both AUs and affects are brief and last approximately for milliseconds or seconds. A total of 7.834 visible muscular movements (AU) were coded, corresponding to 1.805 facial affective expressions. The retest reliability for EMFACS has been reported to vary from 0.89 to 1.00 and intercoder agreement from 0.87 to 1.00 [5]. The validity has been confirmed in ethnological and ethological studies [23, 24]. In the present study two independent coders at the University of the Saarland in Saarbrücken, Germany, performed the coding. They did not have command of the language spoken in the interviews since they had a different language background than the patients and the interviewers and were blind to the diagnoses of the patients. All interviews for each patient were coded by the same coder. As a reliability check, a sample of the interviews (6 minutes of interview time) was presented to both coders who scored an excellent intercoder agreement (*κ* > 0.80) on rating AU.

#### 2.2.4. Statistics

The statistical package SPSS, version 15, was used. Descriptive statistics were used to summarise the frequency and variation of the facial affects displayed. Within-subjects ANOVAs were performed to test whether the amount of affects varied across the interviews. The affects served as dependent variable with interview as the within-factor having three levels (interview A_1_, interview B_1_, interview C_1_). Wilcoxon tests for repeated measures were employed to test whether the patients displayed identical affects for both interviewers. The independent variable was the interviewer and the depended variable the affects shown by the patients. Finally Mann-Whitney *U* test was used to compare the interviewers' facial affectivity.

## 3. Results

**Figure 1:**
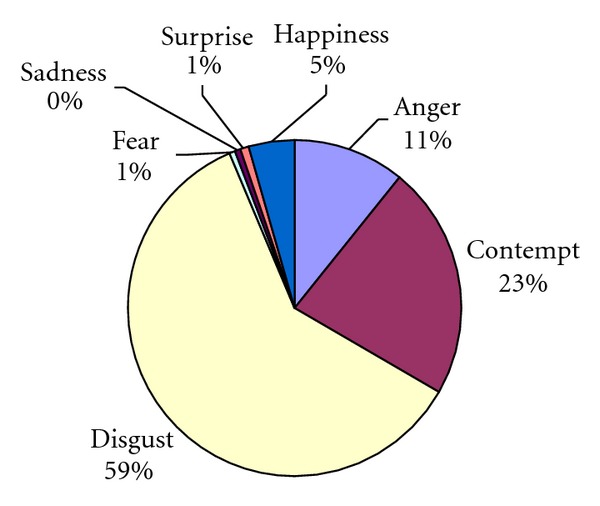
Percentage of patients' facial affective expressions in 24 interviews (interview A_1_, interview B_1_, interview C_1_) with interviewer 1.

**Figure 2:**
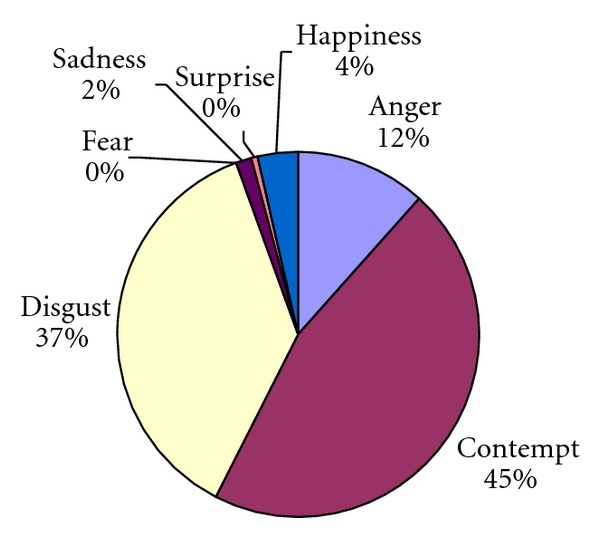
Percentage of patients' facial affective expressions in 8 interviews (interview A_2_) with interviewer 2.

Overall, facial affectivity in patients was predominantly negative. Disgust, contempt and anger predominated (see Figures 1 and 2 and Table 2 for descriptive statistics).

**Figure 3:**
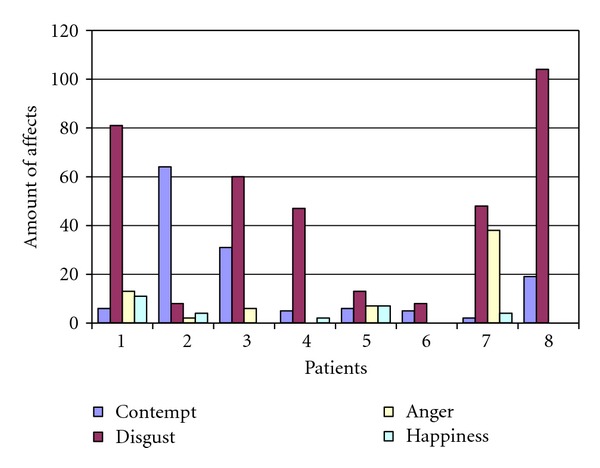
Amount of affects per patient (*N* = 8) in interviews A_1_, B_1_, C_1_.

As the standard deviation in Table 2 shows, there was considerable variation among patients regarding the frequency of affects. Furthermore a closer examination of the patients' affects in data from interviews A_1_, B_1_, and C_1_ reveals that there was a considerable range in total affects displayed (see Figure 3) contributing to the high standard deviations. However, all patients displayed mostly negative affects.

The ANOVAs of data from the 24 interviews (interview A_1_, interview B_1_, interview C_1_) with interviewer 1 revealed no significant effect among patients for any affect, (all *F*_(2,14)_ ≤ 2.246, *P* ≥ 0.143) indicating that the amount of facial affectivity displayed by the patients did not vary throughout the interview series. Error variance for the patients' four most frequent affects was, anger (*F*_(2,14)_ = 0.640), contempt (*F*_(2,14)_ = 1.103), disgust (*F*_(2,14)_ = 0.585), and happiness (*F*_(2,14)_ = 2.246).

**Figure 4:**
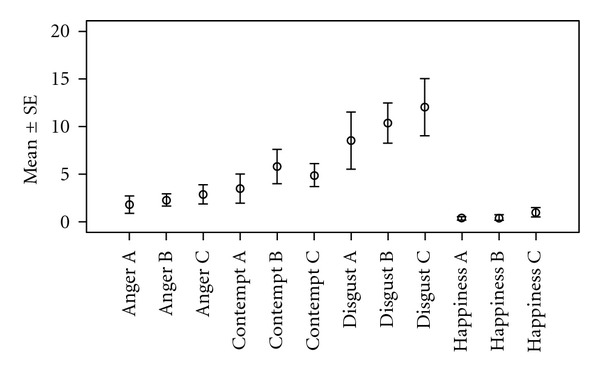
Average number of affects for the patients in 24 interviews (interview A_1_, interview B_1_, interview C_1_) with interviewer 1; error bars indicate standard error of the mean.

Figure 4 shows the average number of affects for the four affects most frequently observed in the patients. The error bars indicate standard error of the mean. Happiness was infrequent and not observed in 3 patients, which contributed to a lower variance for this affect. Disgust appears to increase, although not significantly.

 The patients' affects in interview A_1_ and A_2_ were then compared. Although both disgust and contempt dominated the patients' facial affects, the relative frequency differed. For interviewer 2, the patients displayed more contempt than disgust; yet when interacting with interviewer 1 the same patients displayed more disgust than contempt. However, the other affects displayed towards both interviewers did not differ significantly (all *P *≥ 0.18), except for the higher amount of contempt (*z* = 2.207, *N*-Ties = 6, *P* = 0.027, two tailed) shown to interviewer 2. Thus, with the exception of contempt, the second hypothesis was retained.

 Both interviewers' facial affective expressions were also compared with each other in the A interviews. With the exception of sadness, which bordered on significance (*U* = 14.5, *N*_1_ = 8, *N*_2_ = 8, *P* = 0.06, two tailed), there was no significant difference (all *P* ≥ 0.10) *between* the facial affective expressions of the two interviewers.

## 4. Discussion

The patients showed elevated amounts of negative facial affectivity. Patients most frequently displayed either disgust or contempt, followed by anger. These findings are in accordance with previous research which found that negative facial affectivity is to be expected in the facial activity of patients with schizophrenia [5, 7, 18]. However, contrary to previous findings [5] the most frequent affect in the present study was disgust as well as contempt. Due to the smallness of the sample this should be interpreted with cautiousness. Previous research found disgust to be the second most frequent affect after contempt [5].

 Regarding the stability of facial affective expressions we found no substantial and systematic changes in the amount of affects the patients displayed at the various interviews, which suggests that affects appear stable and seem fairly independent of interview occasion. The patients' affects were not altered by the change of interview partner, with the exception that contempt was more frequently displayed with the first interviewer. No conclusions can be drawn about the reason for this finding, since we cannot relate the results to changes in the pathological state of the patients due to lack of corresponding data. Though, we do not believe that this finding is due to differences in the interviewers' facial affective expressiveness, since we also analyzed their facial affectivity and found no significant differences. We then know that their facial affective behaviour is similar to each other.

 Most of the patients in the present study remained on the same antipsychotic medication at both data collections. To our knowledge there are no studies examining the impact of antipsychotic medication on facial *affective* behaviour in schizophrenia. Studies examining the effects of antipsychotic medication on facial expressiveness in general show contradicting results [3, 4, 6, 25, 26]. However, a newly published review claims that diminished expression in schizophrenia is observed independently of medication [10]. Nonetheless, if there is an impact on facial expressiveness, it is improbable that the type of facial affective expression should be selectively affected. While it is possible that the medicated patients may show reduced amounts of facial affective behaviour in comparison with unmedicated patients, it seems unlikely that the quality of affects shown, whether it was disgust or happiness, was affected.

 As the sample was nonrandom and small, caution is advised in generalising the results to the total population of patients diagnosed with schizophrenia. This type of in-depth investigations does not allow big samples thereby limiting the power. On the other hand, the results are strengthened by the repeated measurement procedure with the same patients participating at several occasions. However, a limitation was that interviewer 2 only performed one interview (A_2_) that was compared with interview A_1_. However, since the analysis of interviews A_1_, B_1_, and C_1_ was performed first and showed that facial affectivity in the patients seemed stable over time, it was therefore assumed that interview A_2_ would be representative of a hypothetical series A_2_, B_2_ and C_2_ and could be used alone without jeopardizing validity. Naturally transcription and use of full series would have permitted more robust comparisons.

Another limitation is the use of only two interviewers both female. Facial affectivity may vary across genders. Also the uneven gender distribution among the patients may raise questions with regard to results generalizability since facial expressivity has been found to be lower in healthy males than in females. However, findings in patients with schizophrenia have been contradicting regarding this matter [9] and a newly published review [10] found only 3 studies that have been examining gender differences and emotional expressiveness in schizophrenia. Two of them found women to be more expressive and one found no differences.

Another limitation that may be crucial to assess the stability of facial expressivity is the lack of the patient's symptom profiles and psychopathological state at the moment of the interviews. It is, for example, possible that facial affectivity may vary depending on whether the patients have positive or negative symptomatology (Positive and Negative Syndrome Scale, PANSS). Given the small sample size it was not possible to analyse neither this aspect, nor gender-related interactions between patients and interviewers.

The interpretation of the facial behavior as emotions is based on the EmFACS dictionary by Paul Ekman, that has been updated and validated by two of the authors (J. Merten, J. Spang) during the last 20 years, but not published. However, the authors (J. Merten, J. Spang) have conducted several decoding studies with various combinations of facial expressions and have selected those with high recognition accuracy. Parts of this work are published in Merten (2005) [27].

The facial affective expression of contempt may involve feelings of superiority as well as disdain for others [28]. Disgust may be connected to feelings of aversion, often for body products, but may also be evoked by sociomoral violations [29]. Disgust is an emotion initially connected to evolutionary purposes, as it leads to avoidance of exposure to potentially harmful situations. It can be triggered by offensive sensations, such as smells, tastes, body products, contact with death but also by sociomoral violations [29]. Disgust may also be triggered by observing other people suffering or by their injuries, although this tendency diminishes with close relationship to the person suffering [28]. Being the object for others disgust is connected with shame. During the creation of intimate relationships, disgust is suspended and in the process of establishing personal intimacy individuals share confessions involving experiences or actions that otherwise would have been the source of disgust and avoidance [28, 30].

Facial expressions may be expressions of underlying emotional states. However, it is important to emphasize that there is no isomorphic relation between facial affective expressions and inner emotional states. We may, for example, smile when we are happy but also when we are feeling uncertain or ashamed. Facial affective expressions do also serve social purposes; they communicate social motives and are driven by social intents. They are important tools in the social interaction; they are present since infancy serving communicative purposes [31, 32]. Reduction of facial expressiveness has been found to related to poorer interpersonal relationships having implications in other social domains [10]. Nonverbal behavior and facial affective expressions signals motives such as the readiness to affiliate attack or continue current interaction and are strongly related to the communicative features of the interaction, such as the topic of discussion. Facial affective expressions may also illustrate the affective components in the narrative. Consecutively, the aversive content of the interviews with focus on psychotic experiences may partly explain the prevalence of “contempt” and “disgust,” since those facial displays are connected to the current social context and also to the verbal content of the interaction and the questions of the interview.

 Therefore, the disgust displayed in the present study might be a reflection of the disgust felt for the sometimes awful experiences connected to the patients' sickness and suffering. As low self-esteem frequently can be observed in schizophrenia [33–35], facial affective expressions of disgust and contempt may also be seen as an expression of self-disgust or self-contempt. Low self-esteem, feelings of shame and guilt are frequently found in first-episode psychosis [36]. The connection between low self-esteem and the facial affective expressions of disgust and contempt in schizophrenia could therefore be a possible next step for future research.

To summarise, negative facial affectivity, mainly disgust and contempt, seems to dominate the facial expressions of patients with schizophrenia in clinical interviews while speaking about psychotic experiences. In the present study this finding seems to be a fairly stable feature independent of time and interviewer. Due to the small sample these findings need to be further replicated.

## Figures and Tables

**Table 1:** Patient characteristics.

Gender	Age	Psychosis diagnosed	Schizophrenia diagnosed	Type of schizophrenia	Medication interv. A_1_, B_1_, C_1_	Medication interview A_2_
M	27	1992	1997	Paranoid 295.30	Risperidone	Risperidone
M	40	1987	1989	Paranoid 295.30	Olanzapine	Risperidone
M	32	1991	2000	Undiff. 295.90	Perphenazine	Perphenazine
M	52	1967	1971	Paranoid 295.30	Zuclopenthixol	Zuclopenthixol
M	36	1994	1994	Paranoid 295.30	Olanzapine	Olanzapine
F	48	1985	1985	Paranoid 295.30	Zuclopenthixol	Zuclopenthixol
F	52	1972	1982	Paranoid 295.30	Ziprasidone	None
M	27	1998	1998	Catatonic 295.20	Olanzapine	Olanzapine

**Table 2:** Descriptive statistics of affects for patients (*N* = 8) in interviews A_1_, B_1_, C_1_ and interview A_2_.

		Anger	Contempt	Disgust	Fear	Sadness	Surprise	Happiness
A_1_	Mean	2.63	5.00	14.25	0.25	0.00	0.13	0.63
	SD	5.04	8.21	15.04	0.71	0.00	0.35	0.74
B_1_	Mean	2.25	7.75	14.00	0.38	0.13	0.38	0.88
	SD	2.96	9.92	10.01	0.74	0.35	0.52	1.46
C_1_	Mean	3.38	4.50	17.88	0.00	0.13	0.13	2.00
	SD	5.40	5.40	14.82	0.00	0.35	0.35	2.51
A_2_	Mean	3.63	14.13	11.38	0.00	0.50	0.13	1.63
	SD	3.02	19.42	14.32	0.00	1.07	0.35	3.16
